# A Novel Acoustic Uroflowmetry-Based Mobile App Voiding Diary: Comparison with Conventional Paper-Based Voiding Diary

**DOI:** 10.1155/2022/3390338

**Published:** 2022-04-25

**Authors:** Jung Kwon Kim, Hwanik Kim, Jin-nyoung Ho, Seong Jin Jeong, Sangchul Lee

**Affiliations:** ^1^Department of Urology, Seoul National University Bundang Hospital, Seongnam, Republic of Korea; ^2^Department of Urology, Hallym University Sacred Heart Hospital, Anyang, Republic of Korea

## Abstract

**Objectives:**

To evaluate the usefulness of a novel acoustic uroflowmetry- (UFM-) based mobile application (app) voiding diary (VD) focusing on the (1) compliance and (2) correlation with a conventional paper-based VD.

**Materials and Methods:**

A total of 78 patients were included between December 2019 and June 2020, and a subsequent review of all data was performed. The analyzed data were as follows: (1) survey of convenience/satisfaction/preference comparing the two methods, (2) compliance regarding the completeness of both methods, and (3) correlation of each metric (24-hour urine volume, nocturnal urine volume, nocturnal polyuria index, total number of voids, number of daytime voids, number of nocturnal voids, and maximal bladder capacity) between the two methods.

**Results:**

The survey results of convenience, satisfaction, and preference were as follows. With regard to convenience and satisfaction area, higher scores are reported in the mobile app VD (mean ± standard deviation (SD); convenience: 7.47 ± 2.19 [app] vs. 4.20 ± 2.49 [paper]; satisfaction: 7.36 ± 2.17 [app] vs. 5.07 ± 2.65 [paper]). The median score of the overall preference for using the mobile app instead of the paper-based VD was 9 out of 10 (mean ± SD7.82 ± 2.68). We also found a good correlation between the two methods for nocturnal urine volume (*r* = 0.55, *p* = 0.04), nocturnal polyuria index (*r* = 0.66, *p* = 0.23), total number of voids (*r* = 0.9, *p* = 0.02), number of nocturnal voids (*r* = 0.83, *p* = 0.02), and maximal bladder capacity (*r* = 0.89, *p* = 0.04).

**Conclusion:**

The acoustic UFM-based mobile app VD demonstrated favorable findings compared to the conventional paper-based VD.

## 1. Introduction

COVID-19 is extremely potent and contagious, and we are still feeling the great impact of the global COVID-19 pandemic [1]. Subsequently, most businesses have been forced to shut down as they struggle to contend with the growing number of cases. However, certain essential public services (including hospitals) continue to remain functional with reduced resources [2]. This public health emergency has resulted in a significant redistribution of medical resources and has created a “zero-contact” era to minimize close personal contact [3]. As COVID-19 is prolonged, COVID-related contact restrictions are easing a lot, and the trend of life with COVID-19 has spread, but this varies depending on geographic regions and it is still not easy to live the same as before COVID-19. In the field of urology, hospital access has been allowed only for life-threatening conditions, emergency surgery, and oncological diseases while outpatient visits for benign diseases have been gradually discontinued [4]. The European Association of Urology (EAU) guidelines on male lower urinary tract symptoms (LUTS) and benign prostatic hyperplasia (BPH) have recommended telemedicine for assessment and follow-up in the COVID-19 era, if possible [5]. Recently, Morselli et al. [6] conducted an observational cohort multicenter study on treatment-naïve patients with LUTS or BPH in five urological centers across Europe using “MyBPHCare,” an application (app) including symptom scores and voiding diaries (VDs) for mobile phones. Consequently, they reported the usefulness of the app to avoid contagion for both patients and physicians.

A VD is essential to evaluate both male and female LUTS patients [7]. It contains important clinical information including the volume and timing of the void, related subjective symptoms (such as frequency, urgency, and incontinence), and fluid intake. However, a conventional paper-based VD has several limitations. In the case of incorrect or peculiar data, a conventional paper-based VD would be difficult and unreliable to interpret. Thus, novel methods or devices using electronic data capture, analysis, and interpretation are needed to improve these limitations [8, 9].

In our previous study, we introduced a new smartphone-based uroflowmetry (UFM) device using acoustic analysis and reported results comparable to those of contemporary office-based UFM [10]. Notably, the correlation between voided volume and predicted voided volume was excellent (Pearson's correlation coefficient [PCC, *r*] = 0.96). To the best of our knowledge, there are no other portable or mobile-based VDs that automatically record the voided volume. Previous smartphone-based VD apps were used only to facilitate patient fulfillment or improve compliance [11, 12].

Thus, we evaluated the usefulness of a novel acoustic UFM-based mobile app VD (Healthy Bladder-Voiding Diary from Soundable Health, Inc.), which records voided volume automatically, by comparing it with a conventional paper-based VD in the current study. We specifically focused on (1) convenience/satisfaction/preference, (2) compliance, and (3) correlation between the two methods in male patients with lower urinary tract symptoms suggestive of benign prostatic hyperplasia (LUTS/BPH).

## 2. Materials and Methods

We performed this prospective comparative study after Institutional Review Board (IRB) approval from the Seoul National University Bundang Hospital (approval number: B-1912/585-301) and in accordance with the ethical standards of the 1964 Declaration of Helsinki and its later amendments. We obtained informed consent from all patients who were enrolled in the study. Personal identifiers were completely removed, and the data were analyzed anonymously.

### 2.1. Novel Mobile Acoustic Voiding Diary (Supplemental Figure 1)

The Healthy Bladder is a smartphone-based, automated VD. The app offers automated measurement using the built-in microphone of a smartphone rather than the void amounts in a standard measuring cup. When a patient urinates into a toilet bowl, the app captures ambient sounds generated by the event and the machine learning algorithm infers key voiding parameters from the acoustic signals. Lee et al. [10] conducted a clinical study to validate the baseline technology. In the study, we described our acoustic uroflowmetry system as follows: with a wireless, smartphone-based approach, the sound data was recorded in real-time with a smartphone application. Sound features were analyzed through audio processing, signal preprocessing, and spectrum analysis. Prediction models were applied to calculate urine flow and parameters. After postprocessing the data for accuracy, voiding parameters of uroflowmetry were generated. In addition to the sound analysis algorithm, pre- and postprocessing refinements to enhance accuracy were added to remove short-term artifacts and outliers, to calibrate background noise levels, and to remove specific noise bands [10].

The app was devised to alleviate the inconvenience and hassle of using a traditional measuring cup and a paper-based voiding diary. Furthermore, it is expected that the app would improve compliance with keeping a VD, guarantee the integrity of the data, and reduce potential recall bias of the patients.

### 2.2. Study Design and Population

A total of 113 patients were included and screened between December 2019 and June 2020 in the current study. Our initial estimated sample size was 35 with a power of 0.9, 5% type 1 error, and noninferiority margin of 0.34, when referring to our previous study [10]. Assuming a 25% of dropout rate, incomplete study, or sound quality problems, 50 patients were recruited as volunteers. However, the authors recruited 113 people who were willing to participate in the study because there was no restriction for participation even if they had reached the targeted number of study subjects under IRB permission. Among them, 35 patients were excluded because they withdrew informed consent (*n* = 5), were female (*n* = 5), did not complete both VDs (*n* = 13), or had no app-supported smartphone (*n* = 12). Finally, 78 patients with adequately recorded 3-day voiding diary by both methods were enrolled, and a subsequent review of all data was performed (Supplemental Figure 6).

The analyzed data was as follows: (1) survey of convenience/satisfaction/preference comparing the two methods, (2) compliance regarding the completeness of both methods, and (3) correlation of each metric (24-hour urine volume, nocturnal urine volume, nocturnal polyuria index, total number of voids, number of daytime voids, number of nocturnal voids, and maximal bladder capacity) between the two methods.

### 2.3. Survey

To eliminate recall bias, the patients completed a questionnaire at the end of each study. The three questions in the questionnaire were as follows: Q1: how convenient was the mobile app VD compared to the conventional paper-based VD (convenience)? Q2: how satisfied were you with the mobile app VD compared to the conventional paper-based VD (satisfaction)? Q3: do you prefer the mobile app VD to the conventional paper-based VD (preference)? The ratings for all three questions were separately self-reported on a scale of 0 (negative) to 10 (positive).

### 2.4. Statistical Analysis

Independent *t*-tests and equal-variance tests were used to determine whether there was statistical evidence that the associated means and variances of the two methods, the conventional paper-based VD and the acoustic UFM-based mobile app VD, were significantly different. To validate the statistical characteristics of the voided volume from each method, independent *t*-tests and equal-variance tests were also chosen for the associated means and variances, respectively. The statistical analysis and calculations were performed using the Python™ v3.6.9 programming language and its scientific computing package SciPy v1.4.1 (Python Software Foundation, Beaverton, OR, USA).

## 3. Results

### 3.1. Baseline Characteristics of Total Patients

A total of 78 male LUTS/BPH patients who completed both the conventional paper-based and acoustic UFM-based mobile app VDs for at least three days each were analyzed in the study. The median age and body mass index were 55.0 years (interquartile range [IQR], 40.0–65.0) and 24.8 kg/m^2^ (IQR, 23.9–26.6), respectively. The median international prostate symptom score and overactive bladder symptom score were 13 (range, 1–31) and 4 (range, 1–11), respectively. The median total prostate volume and transitional zone volume measured by transrectal ultrasonography was 32.0 mL (IQR, 25.0–40.6) and 12.3 mL (IQR, 7.4–16.6), respectively. The median prostate-specific antigen value was 0.951 ng/mL (IQR, 0.585–1.820).

### 3.2. Compliance and Survey Results

The completeness results of each method are described in Table 1. The acoustic UFM-based mobile app VD showed more completed entries that included 1-day full, wake-up time, and bedtime voids than the conventional paper-based VD (all, *p* < 0.05). In addition, among 78 patients, more than 85% (*n* = 67) completed more days on the VD app than the paper-based VD. In contrast, the VD app had fewer total voids recorded. However, the statistical characteristics of both methods were similar. This means that the difference was due to not only the technical differences between the two methods but also the intrinsic deviation of the statistic number of voids and/or voided volume (Supplemental Figure 2).

**Figure 1:**
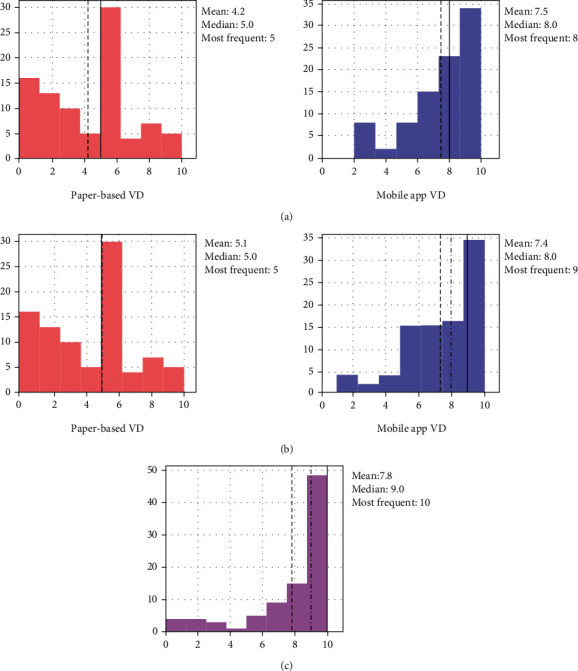
Survey results of (a) convenience, (b) satisfaction, and (c) preference.

The survey results of convenience, satisfaction, and preference are depicted in Table 2 and Figure 1. As for convenience and satisfaction area, higher scores are reported in the mobile app VD (mean ± standard deviation (SD); convenience: 7.47 ± 2.19 [app] vs. 4.20 ± 2.49 [paper]; satisfaction: 7.36 ± 2.17 [app] vs. 5.07 ± 2.65 [paper]). On a scale of 0 to 10, the ratings for all three questions were higher than 8 for the acoustic UFM-based mobile app VD. The median score of the overall preference for using the mobile app instead of paper-based VD was 9 out of 10 (mean ± SD7.82 ± 2.68, Figure 1).

### 3.3. Correlation between Conventional Paper-Based and Acoustic UFM-Based Mobile App VDs

Each VD method was performed individually, so the two sets of VD methods were kept in a separate manner and would not represent the same voids over time. We also recognized the daily variation in voided volume and the number of voids for each patient (Supplemental Figure 3). Therefore, it can be assumed that the direct comparison of the two methods represented different events with no concurrency, which could be an unreliable way to validate the acoustic UFM-based mobile app VD. Thus, we performed further analysis as follows.

The VDs of 30 of the 78 participants passed the independent *t*-test, and the VD of 51 patients passed the equal-variance tests. That is to say, 30 pairs of paper-based and app-based VDs were not significantly different in terms of voided volume, and 51 pairs were not significantly different in variances in voided volume. Subsequently, 24 pairs of VDs passed both the associated means and variance tests simultaneously (Supplemental Figure 4). For the 24 participants, with a significance probability of 11.0%, we could not reject the null hypothesis and concluded that the mean voided volume of the paper-based and app-based VDs was not significantly different. Similarly, through the equal-variance test, since the significance probability was 61.0% and higher than our chosen significance level of 0.05, we concluded that the variance in the voided volume of the two methods was not different. Accordingly, we also performed a subgroup analysis of these 24 patients separately.

**Figure 2:**
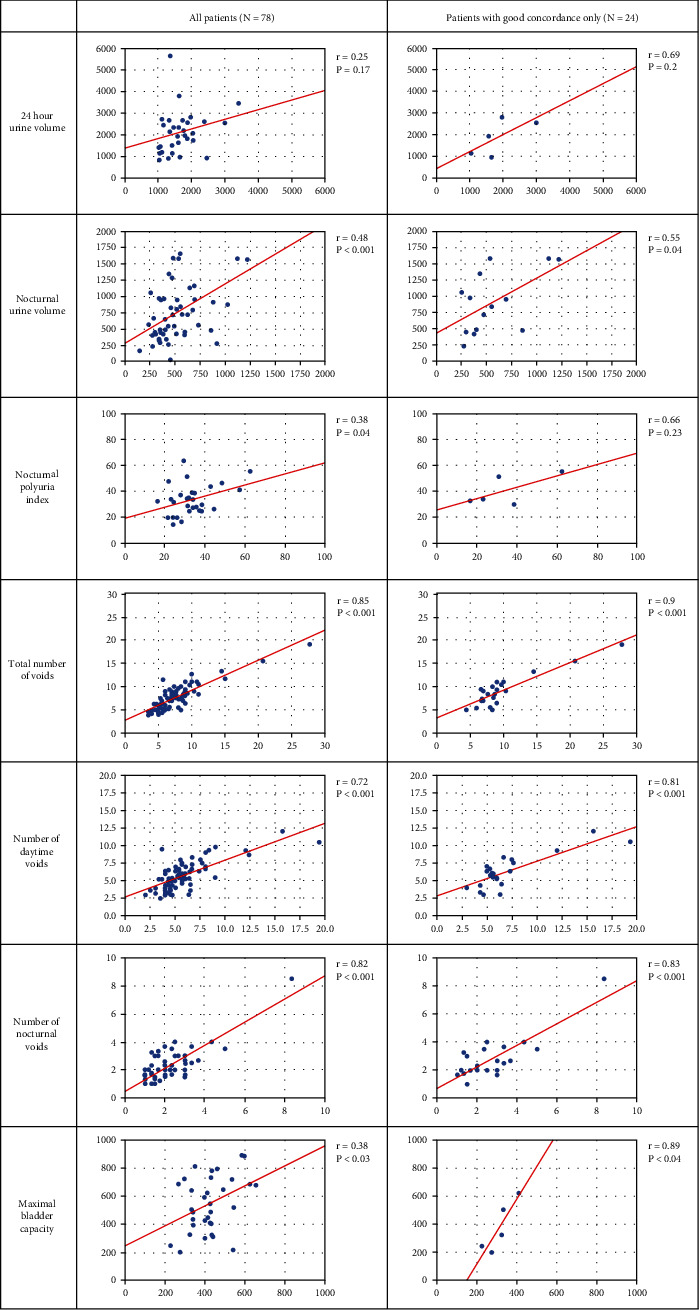
Scatter plot demonstrating the correlation between conventional paper-based and acoustic uroflowmetry-based mobile app VD data regarding each metrics.

A scatter plot demonstrating the correlation between the two methods is shown in Figure 2. A good correlation was observed between the two methods for nocturnal urine volume (*r* = 0.55, *p* = 0.04), nocturnal polyuria index (*r* = 0.66, *p* = 0.23), total number of voids (*r* = 0.9, *p* < 0.001), number of nocturnal voids (*r* = 0.83, *p* < 0.001), and maximal bladder capacity (*r* = 0.89, *p* = 0.04) in 24 patients with good concordance (Figure 2).

### 3.4. Correlation between Conventional Paper-Based and Acoustic UFM-Based Mobile App VDs according to Mobile Platforms: Android and iOS

The VDs of 28 out of 51 participants who used Android smartphones and 12 out of 27 participants who used iPhones passed the Kolmogorov-Smirnov test, which was used to quantify the statistical equality of the paper-based and app-based VD methods. Forty pairs of paper-based and app-based VDs were not significantly different in terms of voided volume (Supplemental Figure 5). For the 28 and 12 participants with Android and iOS smartphones, respectively, with significance probability ranging from 0.05 to 0.94 and 0.19 to 0.99 for Android and iPhone users, respectively, we could not reject the null hypothesis and concluded that the mean voided volume of the paper-based and app-based VDs was not significantly different.

## 4. Discussion

Conventional paper-based VDs have several limitations including patient fulfillment/compliance and time-consuming interpretation of the data. Accordingly, electronic VDs designed to optimize compliance and effectiveness have been introduced. Compu-Void, a personal computer-based VD, was the first electronic VD model. Patients could use it after downloading programs onto a personal computer. Rabin et al. [13] found significantly high patient compliance and information accuracy with this device. Since then, research on this has been actively conducted. Mateu et al. [11] developed a 3-day mobile app VD for smartphones (eDM3d). It consisted of the main interface with four buttons (wake up, go to bed, urinate, and drink) and recorded and automatically transferred the data to an internet server to obtain an electronic report. Consequently, they found that eDM3d was a useful tool easily filled in by patients with a high satisfaction rate. Sussman et al. [14] conducted a randomized trial comparing a web app (BladderTrakHer) developed by the American Urogynecologic Society to the conventional paper-based VD for reliability and satisfaction. They found that BladderTrakHer and the paper-based VD had good test-retest reliability, although the number of voids and leaks entered was slightly lower for the electronic VD (28.0 vs. 25.5 [*p* = 0.03] and 4.5 vs. 2.8 [*p* = 0.02], respectively). However, in these previous studies, apps were used only to facilitate patient fulfillment or improve compliance [11–14].

To the best of our knowledge, our acoustic UFM-based mobile app VD was the first portable device recording voided volume automatically. In our previous study, we introduced a smartphone-based UFM device using acoustic analysis and reported a result comparable to that of contemporary office-based UFM [10]. The results showed an excellent correlation between acoustic and standard UFM with regard to maximum and average flow rates as well as voided volumes. In the current study, we expanded the research on VDs and again confirmed the accuracy of the acoustic method. The current novel acoustic UFM-based mobile app VD could also provide longitudinal trends in the urodynamic parameters in a quantitative manner, which will be useful for healthcare providers and payers who need to prescreen and monitor LUTS/BPH patients [15–17]. The possibility that these data may be collected remotely outside the office setting will be valuable as healthcare trends to remote care [1–4, 18]. In addition, the daily use of the mobile app VD will be valuable in monitoring the patient's response to therapy. The time to void and the voided volume can be calculated and entered by the predicted urine flow from each recorded voiding event and automatically consolidated for each day. This quantitative and ease-of-use app might improve shortcomings of current VDs such as incomplete VDs with missing values and low compliance [19–21].

The limitations include the interference of results with significant background noise. In addition, we only included male LUTS/BPH patients due to the gender differences in acoustic UFM techniques. Male patients have louder voiding sounds derived from the standing position, which has no barrier to noise reduction. Therefore, more sophisticated and separate methods are required for each gender. In addition, the voided volume recorded in the acoustic UFM-based mobile app VD was generally larger than that in the paper-based VD (Supplemental Figures 4 and 5). Thus, more accurate measures of urine volume in the VD app are needed to improve the concordance of both methods. As this is an intrasubject comparison study of two measurement methods, the authors sincerely compared 2 methods from questionnaires filled with a series of identical questions, especially about convenience and satisfaction. Nevertheless, a question about preference was a single question of the quantitative figure (not just a simple-dichotomous question like “which do you prefer, mobile or paper voiding diary?”) to reveal the delicate preference of individual subjects. This is one of the major limitations of the study in that authors could not fully evaluate intrasubject variability which might weaken our significant results. With the technical advancement of accurate volume measurements, the evaluation of a larger number of cases is expected to be statistically significant in both methods.

## 5. Conclusions

The acoustic UFM-based mobile app VD demonstrated favorable findings in terms of convenience/satisfaction/preference and compliance and also showed reliable correlation with a conventional paper-based VD in male LUTS/BPH patients. Future large-scale prospective studies are needed to further validate our results.

## Figures and Tables

**Table 1:** Comparison of compliance between conventional paper-based and acoustic uroflowmetry-based mobile app voiding diaries (total *N* = 78).

Completeness	Conventional paper-based VD	Acoustic UFM-based mobile app VD	*p*
1-day full, days (mean ± SD)	372 (4.13 ± 0.78)	401 (4.46 ± 1.38)	0.0453
Wake-up time, days (mean ± SD)	301 (3.34 ± 1.32)	375 (4.17 ± 1.46)	0.0001
Bedtime, days (mean ± SD)	245 (2.72 ± 1.2)	306 (3.4 ± 1.27)	0.0003
Total number of voids, *N* (mean ± SD)	2254 (25.04 ± 11.29)	2125 (23.61 ± 11.22)	0.3941

**Table 2:** Comparison of the survey results between conventional paper-based and acoustic uroflowmetry-based mobile app voiding diaries (total *N* = 78).

		Mean ± SD	95% CI	Ranges
(A) Convenience	Paper	4.20 ± 2.49	3.68–4.72	0–10
	App	7.47 ± 2.19	7.01–7.93	2–10
(B) Satisfaction	Paper	5.07 ± 2.65	4.51–5.62	0–10
	App	7.36 ± 2.17	6.90–7.81	1–10
(C) Preference	—	7.82 ± 2.68	7.26–8.38	0–10

## Data Availability

The authors confirm that the data supporting the findings of this study are available within the article and/or its supplementary materials.

## Supplementary Materials

Supplementary 1 Supplemental Figure 1: acoustic uroflowmetry-based mobile app VD (Healthy Bladder-Voiding Diary from Soundable Health, Inc.). Supplemental Figure 2: variation in voided volume for each patient. Supplemental Figure 3: (A) concordance and (B) discordance of conventional paper-based and acoustic uroflowmetry-based mobile app VDs. Supplemental Figure 4: concordance of conventional paper-based and acoustic uroflowmetry-based mobile app VDs regarding voided volume (*N* = 24). Supplemental Figure 5: Concordance of conventional paper-based and acoustic uroflowmetry-based mobile app VDs regarding voided volume according to mobile platforms: (A) Android (*N* = 28) and (B) iOS (*N* = 12).

Supplementary 2 Supplemental Figure 6: study flowchart.
