# Supplementary material for: A Novel Acoustic Uroflowmetry-Based Mobile App Voiding Diary: Comparison with Conventional Paper-Based Voiding Diary
Source: Biomed Res Int. 2022 Apr 25;2022:3390338. doi: 10.1155/2022/3390338 (PMC9041157; doi:10.1155/2022/3390338)
Supplement: Supplementary 1 — Supplemental Figure 1: acoustic uroflowmetry-based mobile app VD (Healthy Bladder-Voiding Diary from Soundable Health, Inc.). Supplemental Figure 2: variation in voided volume for each patient. Supplemental Figure 3: (A) concordance and (B) discordance of conventional paper-based and acoustic uroflowmetry-based mobile app VDs. Supplemental Figure 4: concordance of conventional paper-based and acoustic uroflowmetry-based mobile app VDs regarding voided volume (N = 24). Supplemental Figure 5: Concordance of conventional paper-based and acoustic uroflowmetry-based mobile app VDs regarding voided volume according to mobile platforms: (A) Android (N = 28) and (B) iOS (N = 12). [file 3390338.f1.pdf]

Supplemental Figure 1. Acoustic uroflowmetry-based mobile app VD (Healthy Bladder-Voiding Diary from Soundable Health, Inc.)

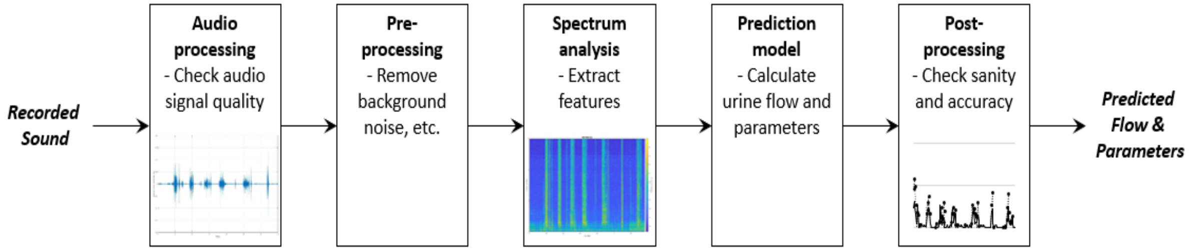

(A) Flow sheet of sound analysis process.

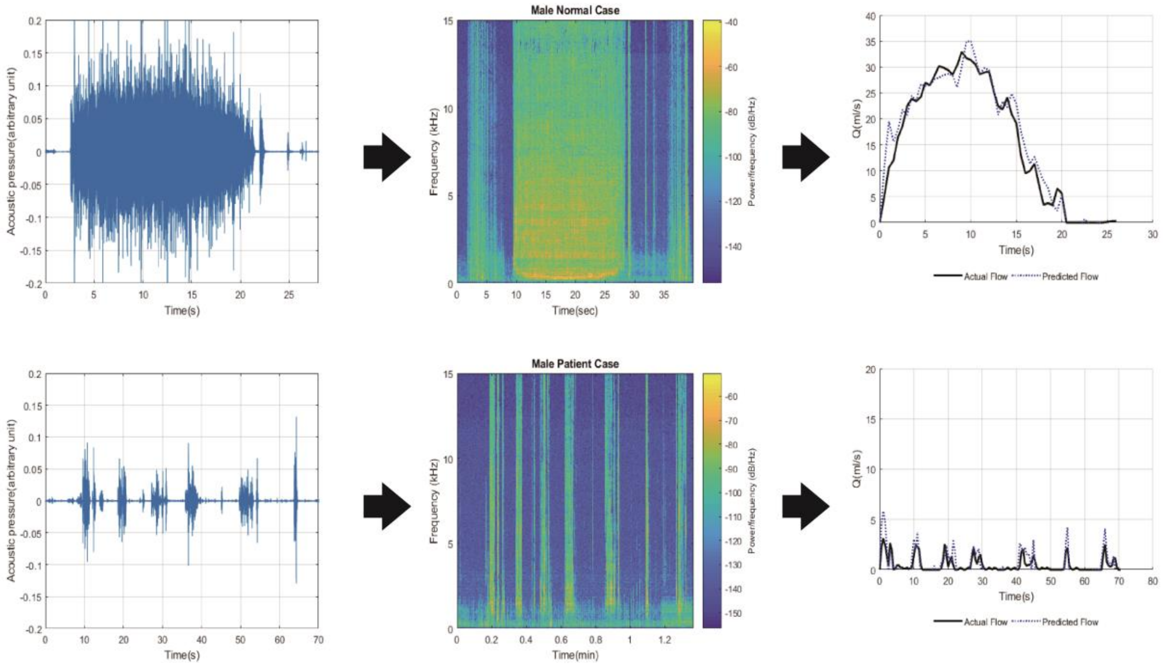

(B) Examples of male acoustic uroflowmetry

Supplemental Figure 2. Variation in voided volume for each patients

A

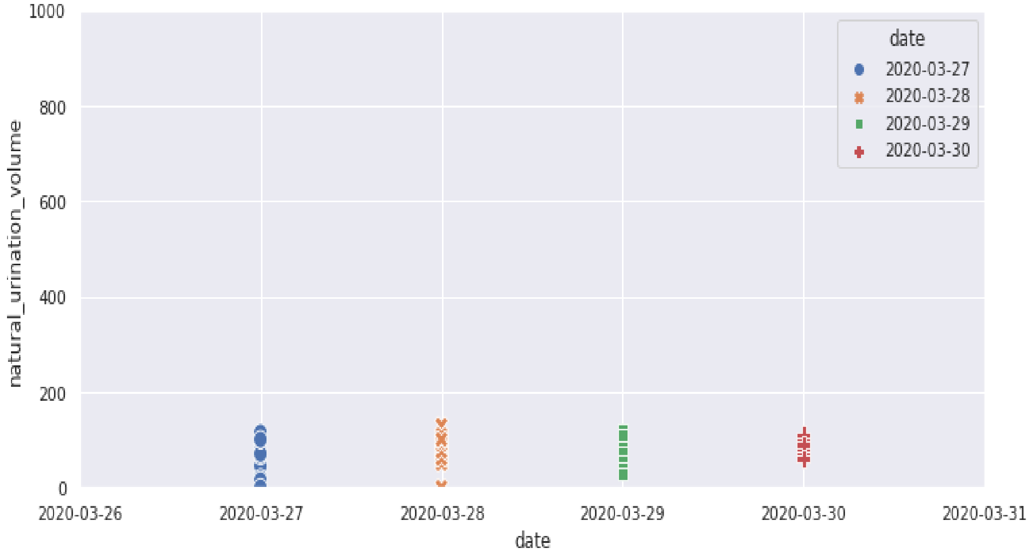

B

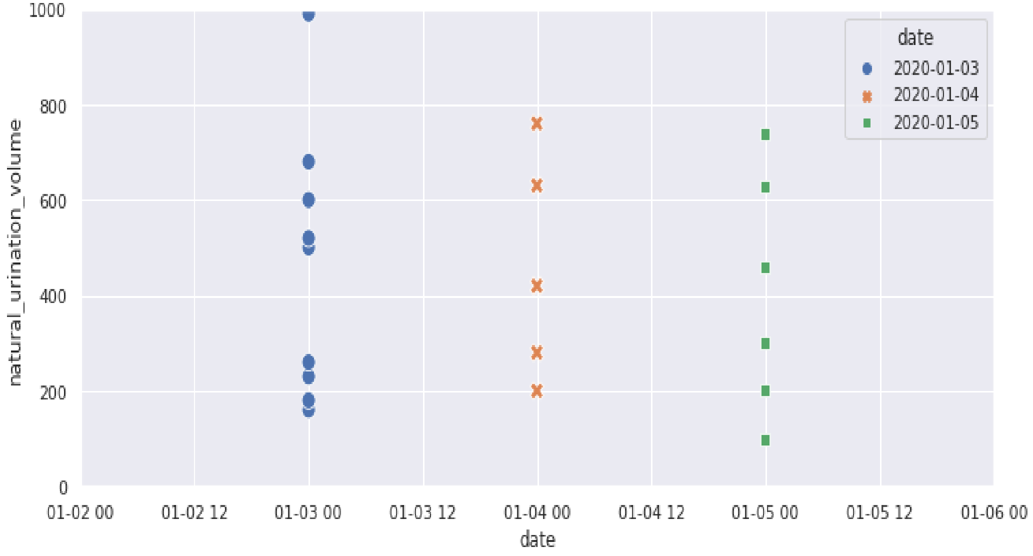

Supplemental Figure 3. (A) Concordance and (B) discordance of conventional paper-based and acoustic uroflowmetry-based mobile app VDs

A

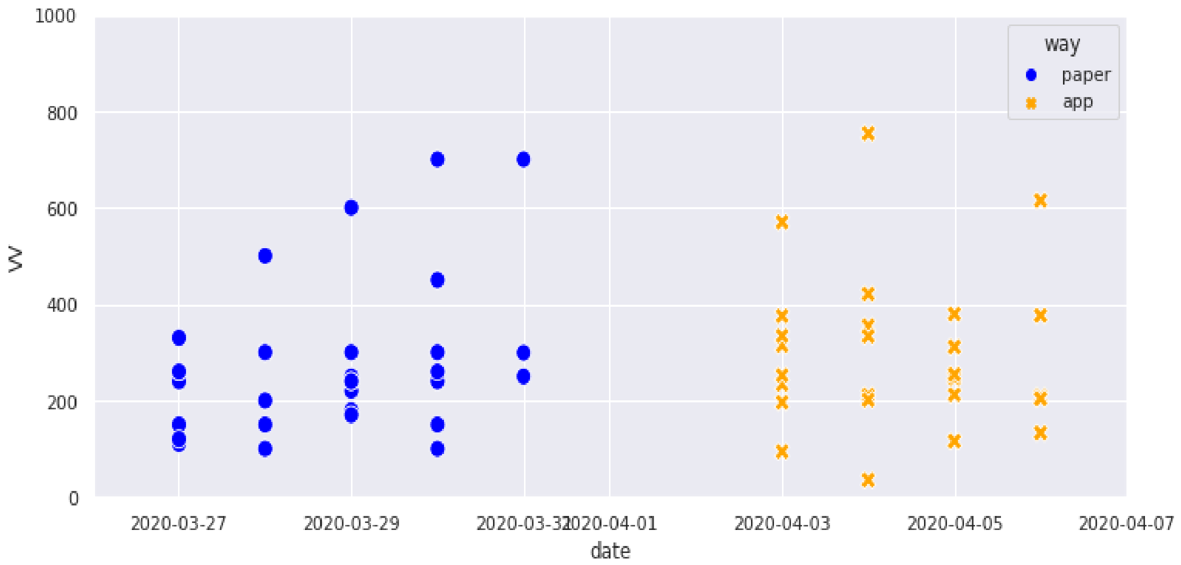

B

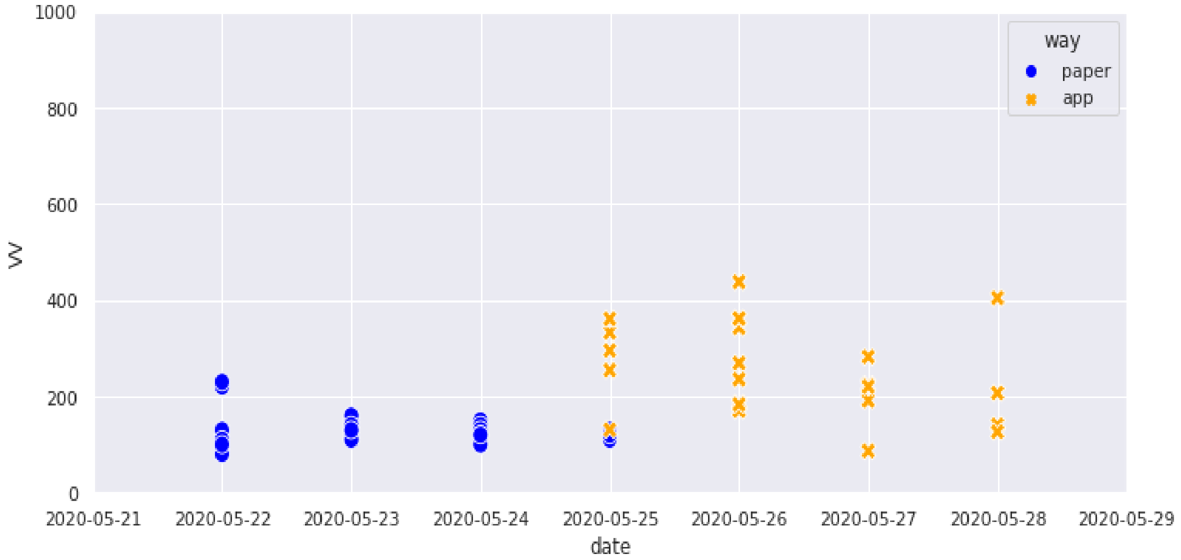

Supplemental Figure 4. Concordance of conventional paper-based and acoustic uroflowmetry-based mobile app VDs regarding voided volume (N=24)

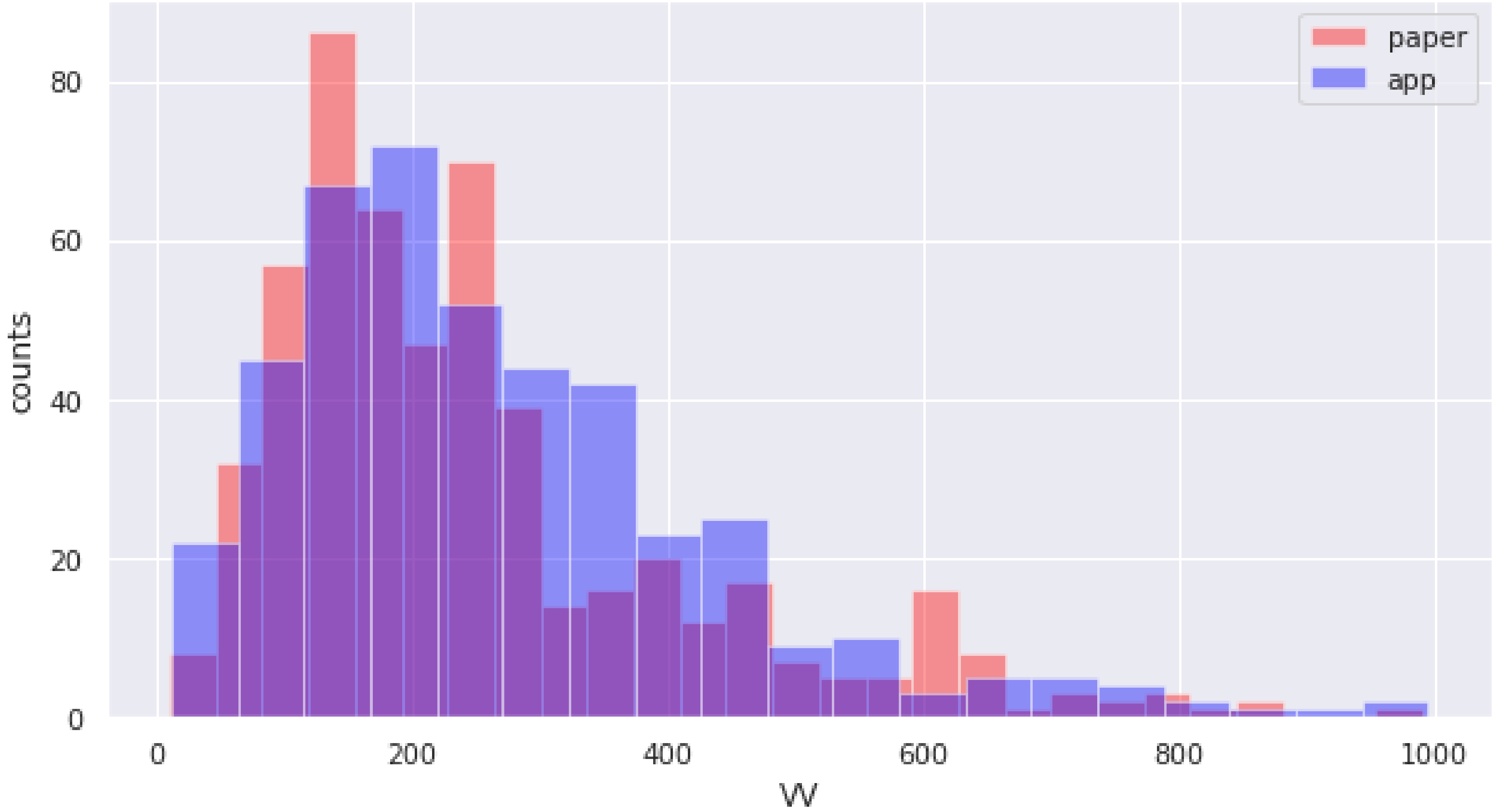

	Independent-two-sample t-test	Equal-variance test
P-value	0.1096	0.6076

Supplemental Figure 5(A). Concordance of conventional paper-based and acoustic uroflowmetry-based mobile app VDs regarding voided volume according to mobile platforms: Android (N=28)

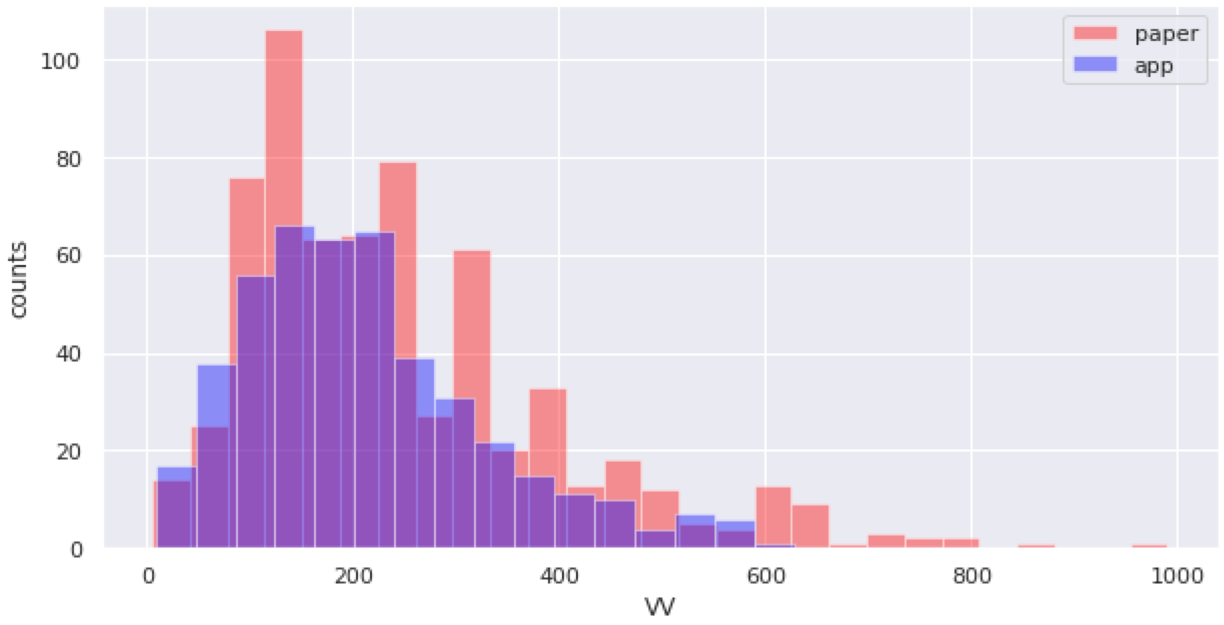

	Kolmogorov-Smirnov Test	Independent-two-sample t-test	Equal-variance test
**P-value**	0.0018	0.5036	0.0005

Supplemental Figure 5(B). Concordance of conventional paper-based and acoustic uroflowmetry-based mobile app VDs regarding voided volume according to mobile platforms: iOS (N=12)

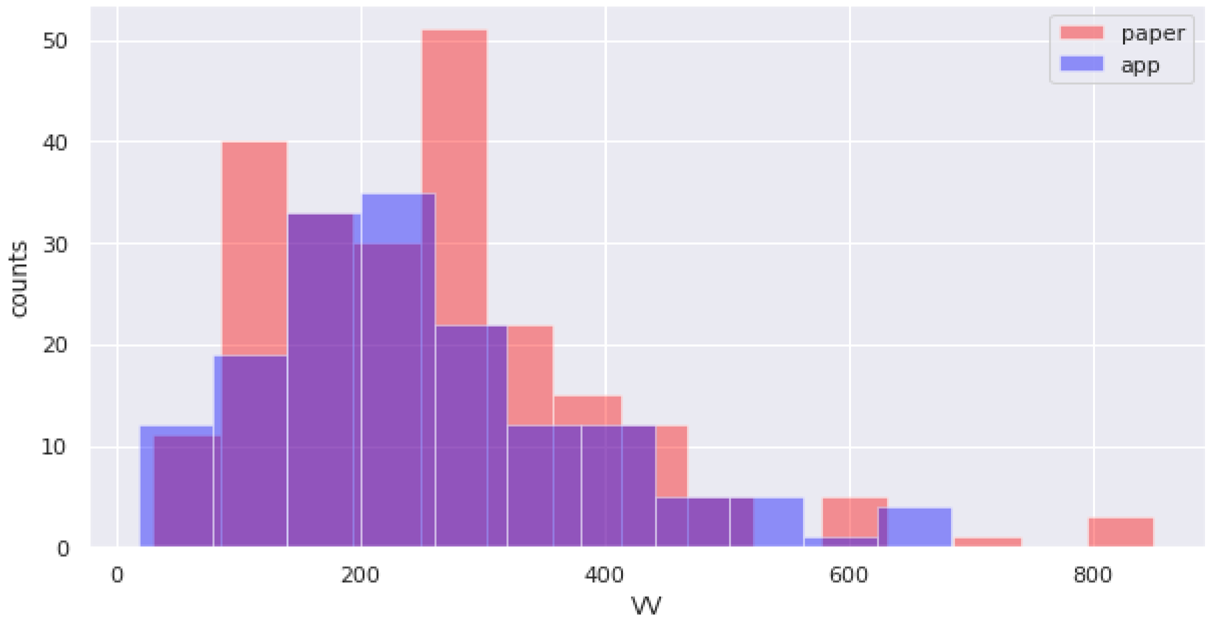

	Kolmogorov-Smirnov Test	Independent-two-sample t-test	Equal-variance test
P-value	0.2189	0.7286	0.6182
