# Supplementary material for: A Novel Acoustic Uroflowmetry-Based Mobile App Voiding Diary: Comparison with Conventional Paper-Based Voiding Diary
Source: Biomed Res Int. 2022 Apr 25;2022:3390338. doi: 10.1155/2022/3390338 (PMC9041157; doi:10.1155/2022/3390338)
Supplement: Supplementary 2 — Supplemental Figure 6: study flowchart. [file 3390338.f2.docx]

**Supplemental Figure 6.** Study flowchart

Assessed for eligibility (n= 113)

♦  Gave consent

♦  Male aged 20 or more

♦  Having voiding symptoms

♦  Can use smartphone and have good literacy

Excluded (n=35)

♦  Declined to participate (n= 5)

♦  Female (n= 5)

♦  Not completed both voiding diaries (n=13)

♦  Not having app-supported smartphone (n=12)

Analyzed (n= 78)
